# Functionalized Carbon Dots from Bio-Based Precursors as Promising Fluorescent Probes for Cancer Cell Imaging

**DOI:** 10.3390/ijms262412185

**Published:** 2025-12-18

**Authors:** Łukasz Janus, Julia Radwan-Pragłowska, Aleksandra Kołodziej-Nowak, Aleksandra Sierakowska-Byczek

**Affiliations:** 1 Department of Biotechnology and Physical Chemistry, Faculty of Chemical Engineering and Technology, Cracow University of Technology, Warszawska 24 Street, 31-155 Kraków, Poland; lukasz.janus@pk.edu.pl (Ł.J.); aleksandra.kolodziej2@gmail.com (A.K.-N.);; 2 CUT Doctoral School, Faculty of Chemical Engineering and Technology, Cracow University of Technology, Warszawska 24 Street, 31-155 Kraków, Poland

**Keywords:** carbon quantum dots, surface modification, circulating tumor cells, fluorescence bioimaging, oncology diagnostics

## Abstract

This study reports the microwave-assisted synthesis and surface modification of carbon quantum dots (CQDs) from natural precursors and their evaluation as fluorescent probes for cancer cell visualization. CQDs were obtained using amino-glucose as the carbon source and betaine, marine collagen, or dopamine as surface modifiers. Further functionalization with 7-amino-4-(trifluoromethyl)coumarin enhanced their fluorescence properties. Spectroscopic analyses confirmed successful surface modification, with coumarin-modified CQDs displaying a strong emission peak at ~500 nm and approximately 1.5-fold higher fluorescence intensity compared to unmodified CQDs. Cytotoxicity testing on MG-63 osteosarcoma cells showed cell viabilities above 80% for selected samples, fulfilling ISO 10993-5 criteria for non-toxicity. In vitro bioimaging of astrocytoma 1321N1 cells demonstrated bright and uniform intracellular staining, confirming effective cellular uptake. Compared with the literature reports of green-synthesized CQDs, our results indicate comparable or superior fluorescence performance and similar levels of biocompatibility. These findings highlight the potential of surface-engineered CQDs as biocompatible nanoprobes for cancer diagnostics and represent an initial step toward their application in the detection of circulating tumor cells (CTCs).

## 1. Introduction

Carbon quantum dots (CQDs) are a unique class of nanomaterials that have attracted significant attention due to their optical properties, biocompatibility, and versatility in a wide range of applications [1,2,3,4]. Typically measuring less than 10 nm in diameter, CQDs consist of a carbon-based core and exhibit strong fluorescence that is primarily influenced by their surface characteristics. The emission of CQDs arises from surface defects or transitions in the forbidden band gap associated with conjugated π bonds within their structure. These features make CQDs promising candidates for bioimaging, sensors, and drug delivery systems.

The optical properties of CQDs can be fine-tuned by altering their surface chemistry, which allows for modification with different biomolecules for targeted applications [5,6,7,8,9,10]. Such surface modifications are essential for enhancing performance, as they improve photophysical characteristics, increase fluorescence quantum yield, and optimize dispersity and stability. Approaches include surface functionalization, heteroatom doping, formation of core–shell structures, and incorporation into composite materials, all of which can expand the range of CQD applications.

Surface functionalization plays a particularly important role in determining both the stability of CQDs in biological systems and their interactions with target molecules. Functionalization with ligands such as polyethylene glycol (PEG), DNA, proteins, or other organic molecules can significantly improve optimize dispersity in water, prevent aggregation, and enhance optical performance [11,12,13,14,15,16,17,18,19,20,21,22,23,24]. Surface passivation also mitigates the negative effects of impurities and reduces surface defects, thereby increasing the number of active sites available for further conjugation. This not only results in enhanced fluorescence but also enables specific interactions with biomolecules, which is critical for biomedical applications such as diagnostic imaging and targeted drug delivery.

One of the most widely applied modification strategies for CQDs is heteroatom doping, in which atoms such as nitrogen, sulfur, oxygen, boron, phosphorus, or fluorine are introduced into the carbon structure [25,26,27,28,29,30]. Heteroatom doping enhances fluorescence efficiency by altering the electronic structure, adjusting the band gap, and creating new energy levels. Among these, nitrogen doping is particularly effective; incorporation of nitrogen atoms generates additional electronic states and reduces the gap between π and non-bonding orbitals, thereby increasing quantum yield. Depending on their configuration—pyridinic, pyrrolic, or graphitic—nitrogen atoms can impart distinct electronic properties, each contributing to improved fluorescence performance. Similarly, sulfur doping modifies the density of states and introduces more emissive traps, which further enhances luminescence. Other dopants, such as boron and fluorine, have also been reported to alter photoluminescence; notably, fluorine doping improves CQD stability across a wide pH range and increases paramagnetic behavior.

Besides heteroatom doping, the choice of synthesis method strongly influences CQD size, structure, and optical properties. Bottom–up techniques such as hydrothermal and solvothermal synthesis are among the most common, producing CQDs with well-controlled size distribution and high structural consistency. These methods are scalable and cost-effective but often require elevated temperatures and long reaction times. To address these limitations, alternative approaches such as microwave irradiation, reduction, and sonochemical synthesis have been explored. These methods offer shorter reaction times, reduced energy consumption, and greater environmental friendliness, while still yielding CQDs with strong fluorescence. Despite such progress, hydrothermal synthesis remains the most widely used approach due to its simplicity, reproducibility, and high yield, consistently producing CQDs with desirable optical properties.

The combination of excellent optical properties, ease of surface modification, and versatility has positioned CQDs as promising candidates for diverse applications, ranging from cancer diagnostics to photodynamic therapy (PDT) and sensor development [22,23,24,25,26,27,28,29,30]. Continued advances in CQD synthesis and functionalization not only expand their biomedical potential but also contribute to the development of sustainable, eco-friendly nanomaterials for medical and environmental technologies.

Among biomedical uses, one of the most exciting directions is cancer diagnostics, particularly the detection of circulating tumor cells (CTCs). CTCs are rare cells shed from primary tumors into the bloodstream and are considered critical indicators of metastasis and poor prognosis [31,32,33,34,35]. Their detection provides valuable insights into tumor dynamics, recurrence, and treatment response. CQDs, due to their strong and tunable fluorescence, can be engineered as highly sensitive probes for labeling, detecting, and potentially isolating CTCs. This capability could substantially improve the accuracy of early diagnosis and open opportunities for personalized cancer therapy.Circulating tumor cells (CTCs) are cancer cells shed from primary or metastatic tumors into the peripheral bloodstream. They are considered key drivers of recurrence and metastasis and are often associated with poor prognosis [34,35,36,37,38,39,40]. Although the precise mechanisms that enable CTC survival in circulation remain incompletely understood, studies suggest that they resist shear stress, evade anoikis and immune destruction, and exhibit resistance to chemotherapy. Consequently, the analysis of CTCs has become a critical area of oncology research, offering opportunities for improved diagnosis, disease monitoring, and personalized therapy.

Several approaches are currently used for CTC analysis. Physical property-based methods exploit differences in size, shape, or elasticity (e.g., microfiltration, microfluidics, and size-based sorting). Surface property-based strategies rely on differential protein expression, with immunomagnetic sorting being a well-established example. Molecular methods, such as PCR, enable the detection of tumor-specific DNA or RNA signatures, while imaging techniques, including optical and fluorescence microscopy, allow direct visualization of CTCs. Each method provides valuable insights, but limitations such as low sensitivity, technical complexity, or high cost hinder their clinical translation [34,35,36,37,38,39,40].

Nanomaterials, and particularly carbon quantum dots (CQDs), have emerged as promising alternatives to overcome these limitations. Owing to their small size, tunable fluorescence, and potential for bioconjugation, CQDs can serve as sensitive probes for labeling, detecting, and studying rare tumor cells in complex biological samples. Preliminary studies have demonstrated the feasibility of using functionalized CQDs for oncology-related CTC diagnostics [40,41,42,43,44,45,46,47,48,49,50].

In this context, our study hypothesizes that surface-engineered CQDs, specifically functionalized with 7-amino-4-(trifluoromethyl)coumarin, can provide enhanced fluorescence while maintaining biocompatibility, thereby enabling effective cancer cell labeling and representing a step toward the development of nanoprobe-based CTC detection tools.

**Figure 1:**
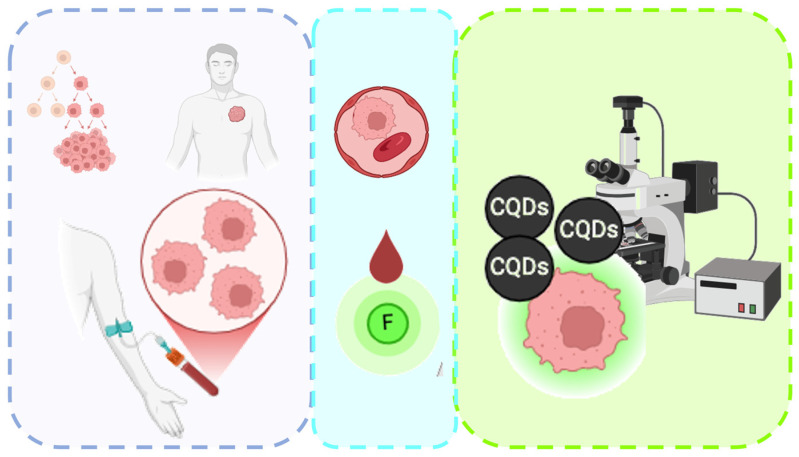
General concept of application of CQDs for CTC detection (blood sample collection, blood incubation with fluorescent CQDs, and in vitro imaging using microscope with epifluorescence adapter).

The objective of this research is to synthesize and characterize surface-modified carbon quantum dots (CQDs) for potential application in oncology diagnostics, with particular emphasis on circulating tumor cell (CTC) detection. We systematically investigate how different synthesis conditions influence the physicochemical and biological properties of CQDs, focusing on their spectroscopic and fluorescence performance. To enhance emission intensity and stability, we functionalize the CQD surface with a coumarin-derived dye. Furthermore, we assess cytotoxicity in cancer cell lines and evaluate the in vitro bioimaging capabilities of the modified CQDs. Collectively, this work aims to determine the feasibility of using surface-engineered CQDs as biocompatible nanoprobes for tumor cell labeling and to establish a foundation for their future application in CTC analysis (Figure 1).

To contextualize our study within the current state of the art, Table 1 summarizes representative reports on carbon quantum dots (CQDs) and related quantum-dot platforms employed for cancer bioimaging and CTC detection. This comparative overview highlights the sources, modifications, fluorescence performance, cellular/CTC contexts, and biocompatibility reported in the literature. Collectively, these studies demonstrate both the potential of surface-modified CQDs for biomedical imaging and the growing role of quantum dots in CTC analysis, while also underscoring the novelty of our approach based on coumarin-functionalized CQDs.

As summarized in Table 1, a number of studies have demonstrated the successful use of CQDs and related quantum-dot platforms for cancer cell imaging, and several reports have highlighted their potential in CTC analysis. However, most existing approaches either rely on synthetic precursors, require complex functionalization steps, or focus on non-carbon quantum dots, which may raise biocompatibility and toxicity concerns. To address these limitations, our work explores a green, microwave-assisted synthesis of CQDs from natural precursors, followed by surface modification with a coumarin-derived dye to enhance fluorescence. By systematically characterizing their spectroscopic properties, cytotoxicity, and cell-labeling performance, we aim to establish coumarin-functionalized CQDs as biocompatible nanoprobes and provide proof-of-concept for their potential use in CTC detection.

## 2. Results

### 2.1. FT-IR Analysis

**Figure 2:**
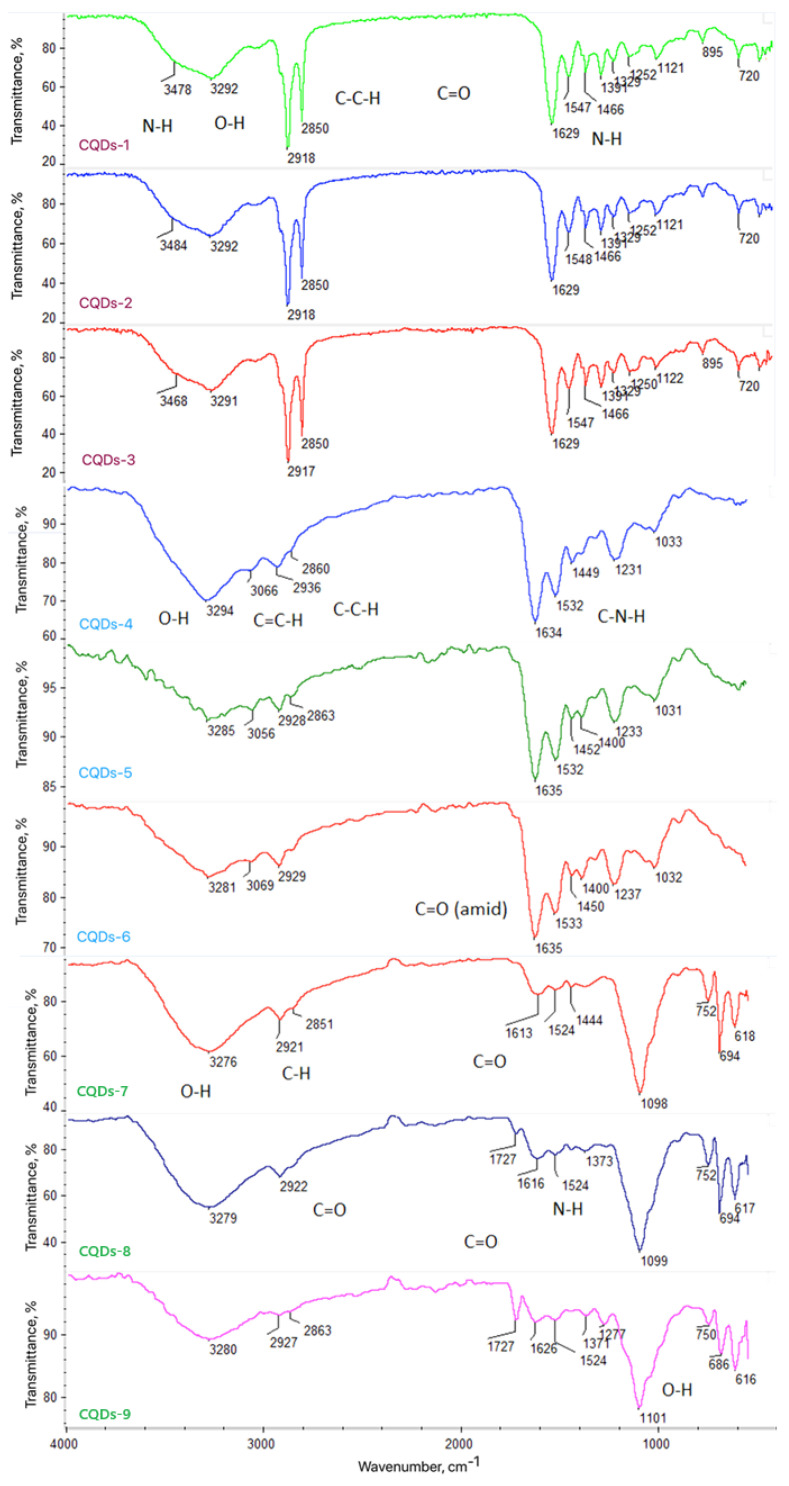
FT-IR spectra of samples (red caption—CQDs modified with betaine; blue caption—CQDs modified with marine collagen; green caption—CQDs modified with dopamine hydrochloride).

The FT-IR spectra of CQDs synthesized from glucosamine and betaine at different microwave irradiation times (CQDs-1–3) confirm the formation of carbon cores decorated with oxygen- and nitrogen-containing functional groups (Figure 2). A broad band at 3480–3290 cm^−1^ corresponds to O–H and N–H stretching vibrations, indicating the presence of hydroxyl and amine moieties, consistent with previous studies on nitrogen-doped CQDs [6,16,25,33]. Bands at 2918 cm^−1^ and 2850 cm^−1^ are assigned to aliphatic C–H stretching, while the strong band at 1629 cm^−1^ reflects C=O stretching of amide or carboxyl groups formed during oxidation [21,26,34]. Additional C–N and C–O vibrations observed at 1500–1300 cm^−1^ confirm heteroatom incorporation within the CQD lattice [22,27]. These functional groups enhance the CQD ability to form suspension and potential biocompatibility [16,20,29]. Subtle spectral shifts with increasing irradiation time indicate progressive oxidation and structural reorganization [33,34].

The FT-IR spectra of collagen-modified CQDs (CQDs-4–6) show characteristic collagen-derived features. The broad band near 3300 cm^−1^ corresponds to O–H/N–H stretching, while strong amide I and II bands at 1634 cm^−1^ and 1535 cm^−1^ confirm peptide bond incorporation [15,16,20,22]. Bands between 1450 and 1030 cm^−1^ correspond to C–N and C–O stretching vibrations typical of protein structures [21,25,27]. Slight spectral shifts suggest that longer microwave exposure increases functionalization and oxidation without altering CQD integrity. Collagen passivation thus enhances hydrophilicity and biocompatibility, as reported for protein-functionalized CQDs [20,22,29,50].

The FT-IR spectra of dopamine-modified CQDs (CQDs-7–9) exhibit bands typical of catechol and amine functionalities. The broad signal at 3280 cm^−1^ indicates O–H/N–H stretching, while bands at 1613 cm^−1^ (C=O/C=C) and 1524 cm^−1^ (N–H bending) confirm dopamine oxidation and amine incorporation [16,20,25,29]. A weak band near 1727 cm^−1^, visible for CQDs-9, suggests partial formation of ester or carbonyl groups after prolonged irradiation [20,22,25,27,33]. These results align with previous findings showing that catechol-based modifiers promote surface passivation, π–π interactions, and enhanced fluorescence [16,20,22,29,50].

Overall, FT-IR analysis confirms successful modification of CQDs with diverse organic molecules, leading to functionalized, biocompatible nanomaterials suitable for bioimaging and sensing applications [15,20,22,29,50,59].

### 2.2. UV–Vis Characterization

**Figure 3:**
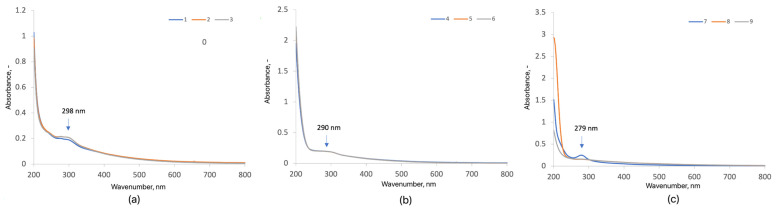
UV–Vis spectra of the samples (3 mg/mL): (**a**) CQDs-1–3; (**b**) CQDs-4–6; (**c**) CQDs-7–9. (Each color represents a different sample).

The UV–Vis absorption spectra of purified carbon quantum dots (CQDs), synthesized via microwave-assisted carbonization of glucosamine and purified by dialysis using a 1 kDa MWCO membrane, are presented in Figure 3. The spectra of aqueous CQD dispersions (0.5 mg mL^−1^) exhibit a pronounced absorption band in the 230–350 nm region, typical of π–π* transitions associated with conjugated sp^2^-hybridized carbon domains [6,16,25,33]. This feature confirms the formation of a graphitic core with delocalized π-electrons. For CQDs-1, CQDs-2, and CQDs-3, absorption maxima were observed at 276 nm (A = 0.201), 277 nm (A = 0.215), and 278 nm (A = 0.215), respectively. The slight redshift with longer irradiation time indicates an increased degree of conjugation and carbonization, consistent with trends reported for time-dependent microwave synthesis of CQDs [33,47,51]. The sharp, well-defined peaks and low baseline scattering after dialysis confirm efficient purification and the removal of unreacted molecular species, similar to other studies employing MWCO membranes for CQD isolation [48,49,52]. Across all synthesized CQDs (CQDs-1–9), absorption maxima were located between 270 and 290 nm, corresponding to π–π* transitions of C=C bonds within the conjugated carbon core. No significant absorption was observed in the 400–600 nm range, indicating the absence of chromophoric impurities or surface defects prior to functionalization [6,47,48,49].

Samples modified with betaine (CQDs-1–3) exhibited absorption peaks at 276–278 nm, while collagen-modified CQDs (CQDs-4–6) showed slightly red-shifted maxima near 290 nm, reflecting extended conjugation due to the presence of amide and hydroxyl groups. Dopamine-modified CQDs (CQDs-7–9) displayed maxima around 279 nm, attributed to π–π* transitions within aromatic catechol domains [16,20,25,50]. These subtle shifts indicate differences in surface chemistry and electronic configuration resulting from the modifiers and reaction conditions. The well-defined absorption features and clean baselines confirm the high optical quality and structural uniformity of the synthesized CQDs. The results are consistent with the literature describing the advantages of microwave-assisted synthesis, which allows for rapid, homogeneous CQD formation with controlled particle size and reproducible optical properties [6,25,33,47,48].

Overall, the UV–Vis analysis confirms that the synthesized CQDs possess a stable conjugated carbon framework and that the observed spectral variations originate from modifier-specific electronic effects rather than impurities. This optical uniformity provides a reliable basis for subsequent fluorescence and bioimaging studies.

### 2.3. Fluorescence

**Figure 4:**
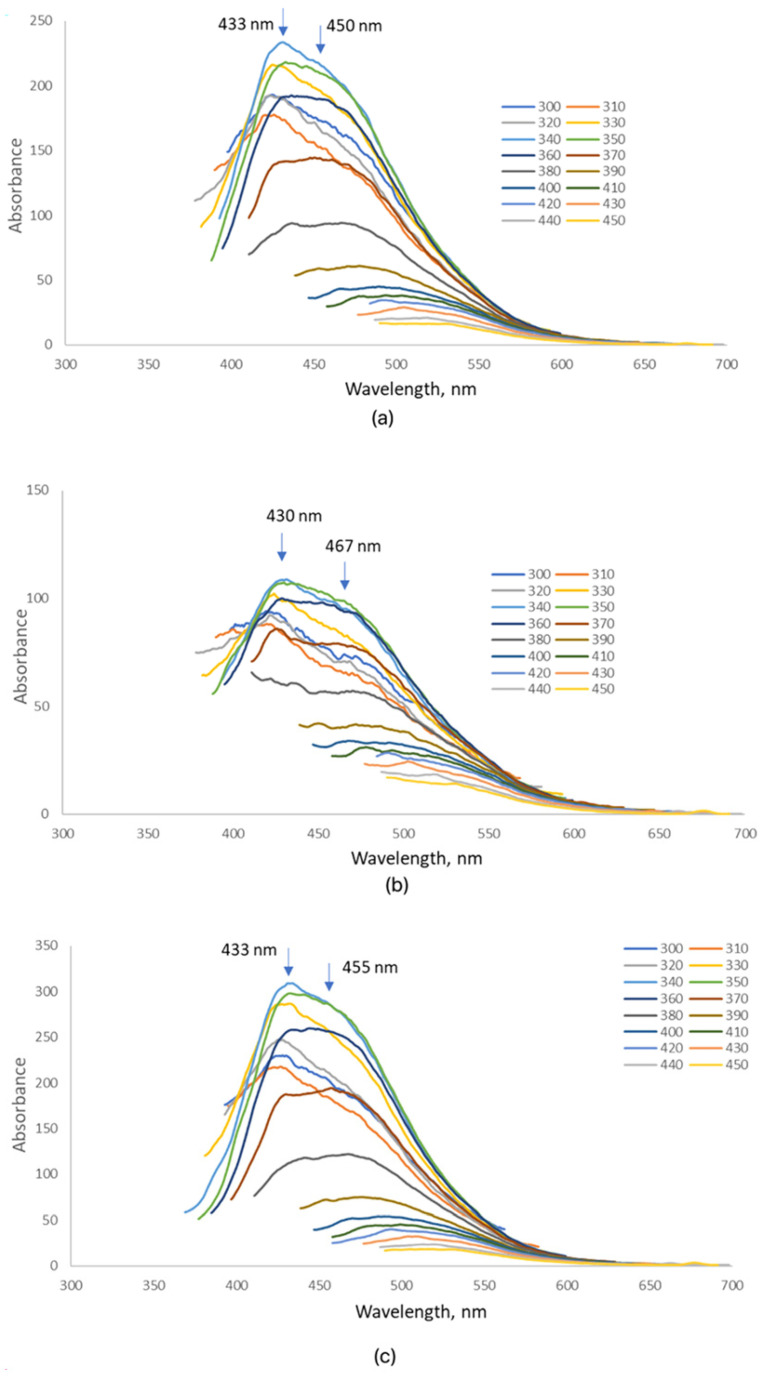
Fluorescence emission spectra of the samples (3 mg/mL): (**a**) CQDs-1; (**b**) CQDs-2; (**c**) CQDs-3 (each color represents a different excitation wavelength).

The fluorescence spectra of betaine-modified CQDs synthesized at different microwave irradiation times (CQDs-1–3) exhibit strong emission in the blue region, with maxima between 430 and 460 nm upon excitation at 360 nm (Figure 4). The most intense peak appeared at 450 nm for CQDs-1, while CQDs-2 and CQDs-3 displayed slightly blue-shifted maxima near 430–440 nm, indicating minor variations in surface structure and oxidation degree. Excitation–emission mapping confirmed the excitation-dependent photoluminescence typical of carbon quantum dots, arising from multiple emissive surface states and heterogeneity in particle size and functional groups [6,16,47,48,49]. The blue emission originates from π–π* and n–π* transitions associated with surface amide and amine functionalities, confirming efficient nitrogen incorporation from glucosamine and betaine precursors [25,50,51]. A moderate redshift with increasing synthesis time suggests gradual surface oxidation and enhanced conjugation. The higher fluorescence intensity of CQDs-1 reflects more effective surface passivation by betaine, which minimizes non-radiative recombination at surface defect sites [33,48,52]. The fluorescence quantum yield (QY), estimated using quinine sulfate as a reference, ranged from 3% to 7%, consistent with previously reported nitrogen-doped CQDs obtained by microwave-assisted synthesis [47,48,50,51]. The absence of additional emission bands or quenching effects indicates high optical purity after dialysis purification. The observed stable, excitation-dependent blue emission confirms that betaine-modified CQDs possess strong fluorescence efficiency and biocompatibility, making them suitable for bioimaging and fluorescence sensing applications [6,16,48,50].

**Figure 5:**
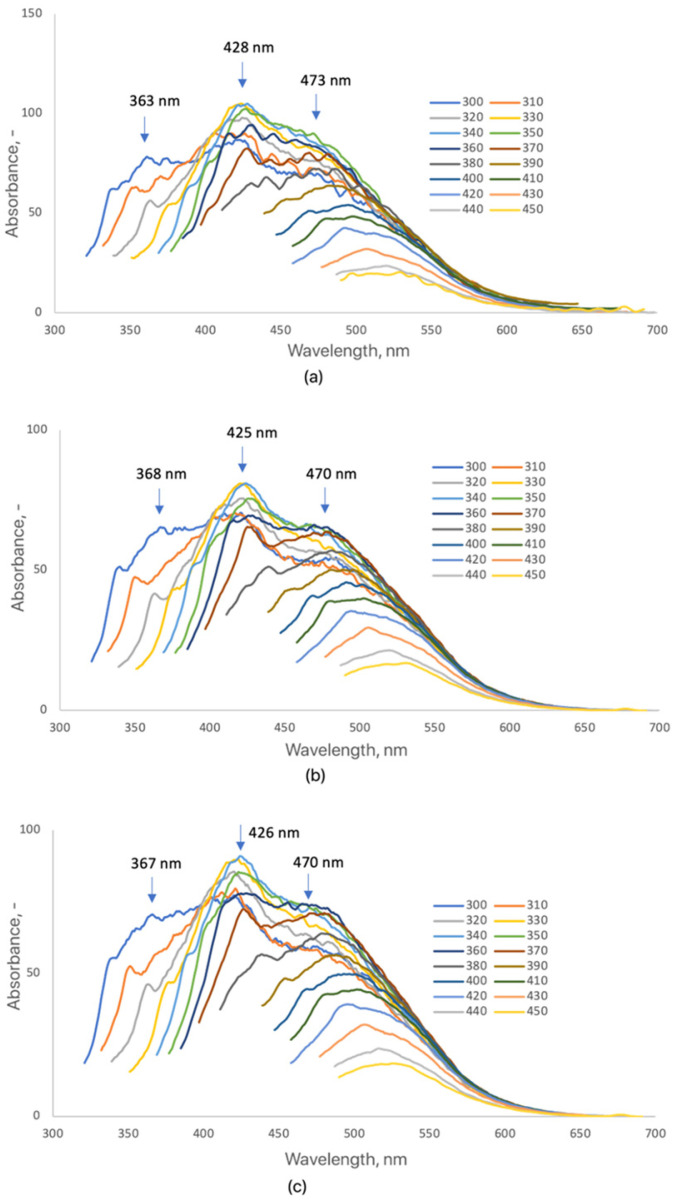
Fluorescence emission spectra of the samples (3 mg/mL): (**a**) CQDs-4; (**b**) CQDs-5; (**c**) CQDs-6. (Each color represents a different excitation wavelength).

The fluorescence spectra of collagen-modified CQDs (CQDs-4–6), synthesized under microwave irradiation for 3, 4, and 5 min, exhibit pronounced excitation-dependent emission profiles (Figure 5). Upon excitation between 320 and 420 nm, multiple emission maxima were observed around 365, 430, and 470 nm, corresponding to transitions from different surface emissive states. These features indicate that marine collagen functionalization significantly influences the photophysical properties of CQDs by introducing amide- and hydroxyl-related energy levels [6,16,20,25]. For CQDs-4, distinct emission peaks at 363, 428, and 473 nm suggest a well-passivated surface with moderate fluorescence intensity. CQDs-5 displayed a red-shifted and more intense emission (368, 429, and 470 nm), reflecting improved conjugation and surface passivation with longer irradiation. CQDs-6 exhibited similar peak positions but slightly lower intensity, consistent with increased graphitization and reduced emissive defect density [47,48,49,50]. The incorporation of marine collagen, rich in amide and carboxyl functionalities, created additional surface states while minimizing non-radiative defects, leading to enhanced fluorescence efficiency and photostability [48,50,51]. The relative quantum yield (QY) of collagen-modified CQDs was estimated at 4–8%, slightly higher than that of betaine-modified analogs, highlighting the beneficial effect of peptide-derived groups on radiative recombination efficiency [16,48,50,52]. Overall, collagen modification effectively tunes the emission wavelength and intensity of CQDs through controlled surface functionalization and passivation, resulting in stable blue-to-green fluorescence suitable for bioimaging and biosensing applications [6,20,48,50].

**Figure 6:**
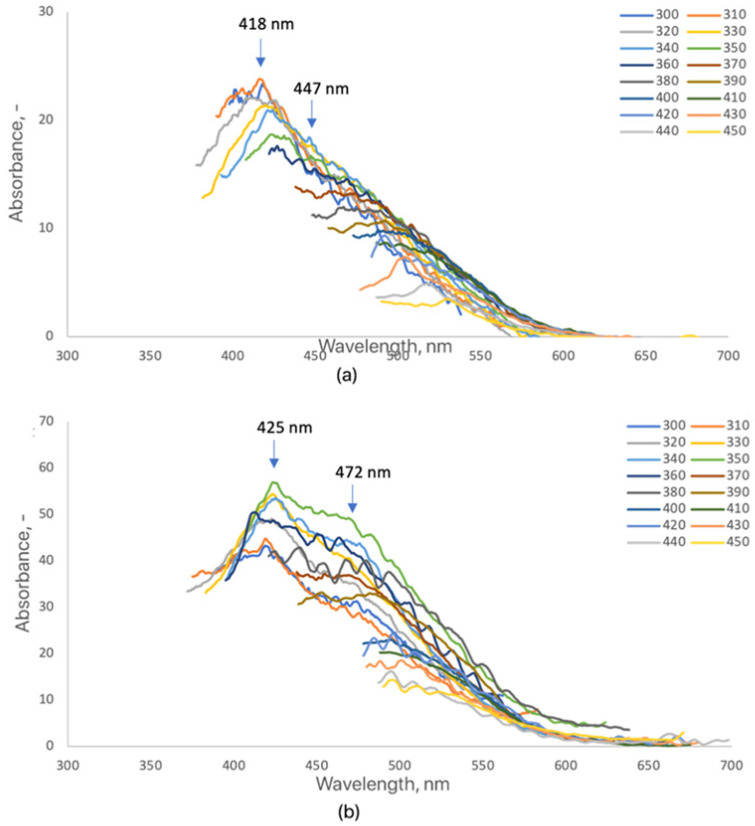
Fluorescence emission spectra of the samples (3 mg/mL): (**a**) CQDs-8; (**b**) CQDs-9 (each color represents a different excitation wavelength).

The fluorescence spectra of dopamine-modified CQDs (CQDs-8 and CQDs-9) revealed distinct emission characteristics strongly influenced by reaction time (Figure 6). The measurements, recorded at excitation wavelengths between 320 and 420 nm, demonstrated excitation-dependent behavior typical for carbon quantum dots with multiple emissive surface states [50,51]. CQDs-8 exhibited two main emission maxima at 418 nm and 447 nm, while CQDs-9 displayed a red-shifted emission at 425 nm and 472 nm, accompanied by higher intensity. The observed redshift and enhanced fluorescence with longer reaction time indicate an increased degree of carbonization and conjugation within the π-electron system, as well as more efficient surface passivation mediated by dopamine-derived amine and catechol groups [52,60]. The appearance of dual emission bands in both samples suggests the coexistence of distinct emissive centers, possibly related to surface defects and the oxidation of dopamine to quinone structures during synthesis [53]. CQDs-7, synthesized with the shortest irradiation time (3 min), exhibited weak or undetectable fluorescence, likely due to insufficient carbonization and incomplete surface functionalization. This observation agrees with previous studies reporting that shorter microwave durations yield poorly emissive CQDs with limited aromatic domain formation [54,60]. The relative fluorescence quantum yield (QY) of dopamine-modified CQDs was estimated at 6–9%, slightly higher than that of collagen-modified analogs, consistent with the enhanced electronic delocalization provided by catechol groups. Such tunable, excitation-dependent blue-to-green emission makes dopamine-functionalized CQDs promising candidates for optoelectronic and bioimaging applications where controlled spectral response is desirable. The CQDs-7 sample displayed negligible emission, with a QY below 1%, indicating incomplete carbonization.

### 2.4. Spectroscopic Characterization After CQD Modification via Cumarin Doping

**Figure 7:**
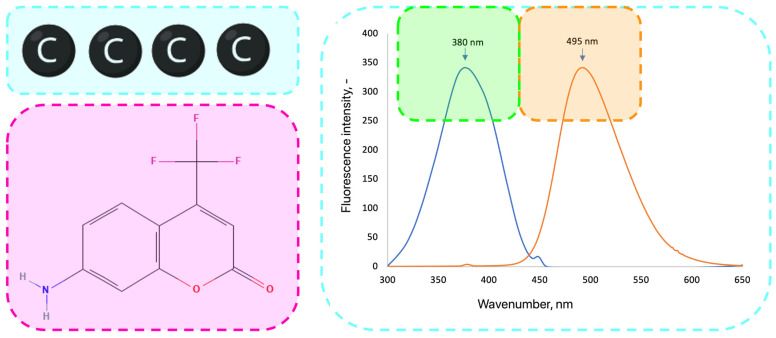
Symbolic visualization of native QCDs (carbon core) and chemical structure of the dye and fluorescence characteristics of 7-Amino-4-(trifluoromethyl)coumarin which was used to modify the CQD surface via formation of covalent bonds to improve spectroscopic characteristics.

The optical properties of carbon quantum dots (CQDs) are highly sensitive to surface functionalization and heteroatom doping, which can markedly enhance fluorescence and expand their application potential. In this study, 7-amino-4-(trifluoromethyl)coumarin, a dye known for its strong fluorescence, extended π–π conjugation, and electron-donating capability, was employed as a dopant to improve the photoluminescence performance of CQDs. Coumarin doping introduces additional emissive states through π–π interactions between the aromatic system of the dye and the conjugated carbon core, facilitating enhanced radiative recombination and tunable emission [16,20,25,50]. This modification strategy has been shown to significantly alter the optical response of CQDs by modifying their surface energy levels and electronic transitions [47,48,51,52]. In the following section, the spectroscopic characterization of coumarin-doped CQDs is presented, including UV–Vis, FT-IR, and fluorescence analyses. These results provide insight into the structural and electronic modifications induced by coumarin incorporation and their effect on the photophysical behavior of the synthesized CQDs (Figure 7) [6,16,48,50].

**Figure 8:**
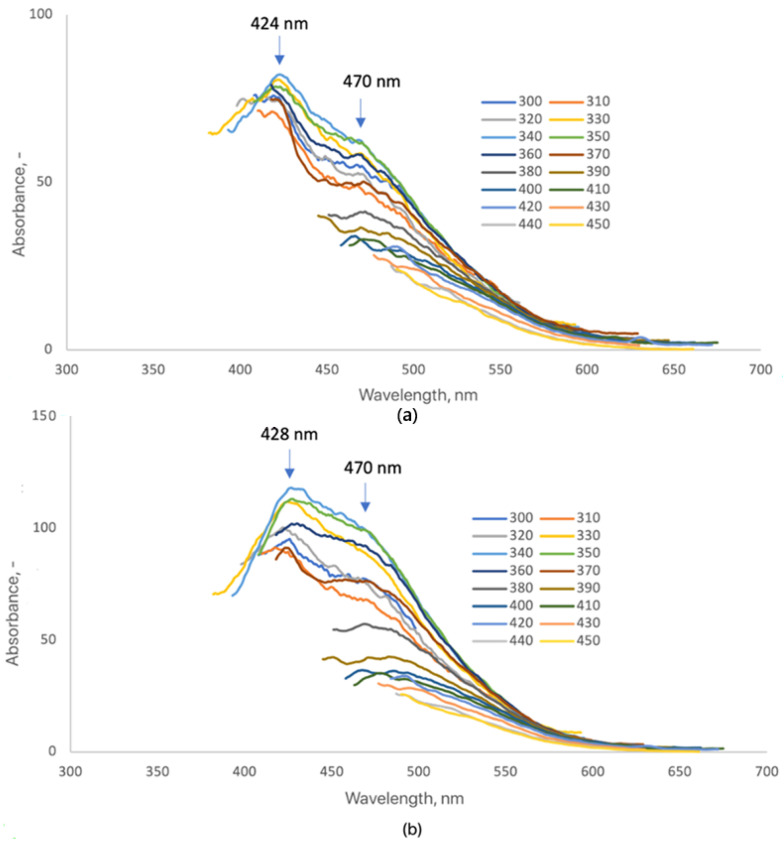
Fluorescence emission spectra of the samples (3 mg/mL): (**a**) CQDs-2-Cum; (**b**) CQDs-3-Cum (each color represents a different excitation wavelength).

Figure 8 shows the fluorescence spectra of CQDs-2 and CQDs-3 after surface modification with 7-amino-4-(trifluoromethyl)coumarin. The modification produced clear spectral changes, confirming successful coupling of coumarin moieties to the CQD surface. Both samples exhibited two distinct emission maxima: CQDs-2 at 424 nm and 470 nm, and CQDs-3 at 428 nm and 470 nm. A pronounced increase in fluorescence intensity relative to unmodified CQDs indicates enhanced radiative recombination and effective surface passivation due to π–π interactions between the coumarin rings and the conjugated carbon domains [16,25,47,48]. The slight redshift in emission maxima suggests the formation of new emissive surface states and stronger electronic coupling between the dopant and the CQD lattice, consistent with earlier studies on dye-functionalized and heteroatom-doped CQDs [20,25,48,50]. Despite these spectral modifications, the characteristic excitation-dependent emission typical for carbon quantum dots was preserved, confirming that their intrinsic photophysical nature remained intact after modification [6,47]. In contrast, CQDs-1 exhibited fluorescence too weak for reliable spectral analysis, likely due to inefficient coupling or partial fluorescence quenching by excess coumarin precursor [48,51].

**Figure 9:**
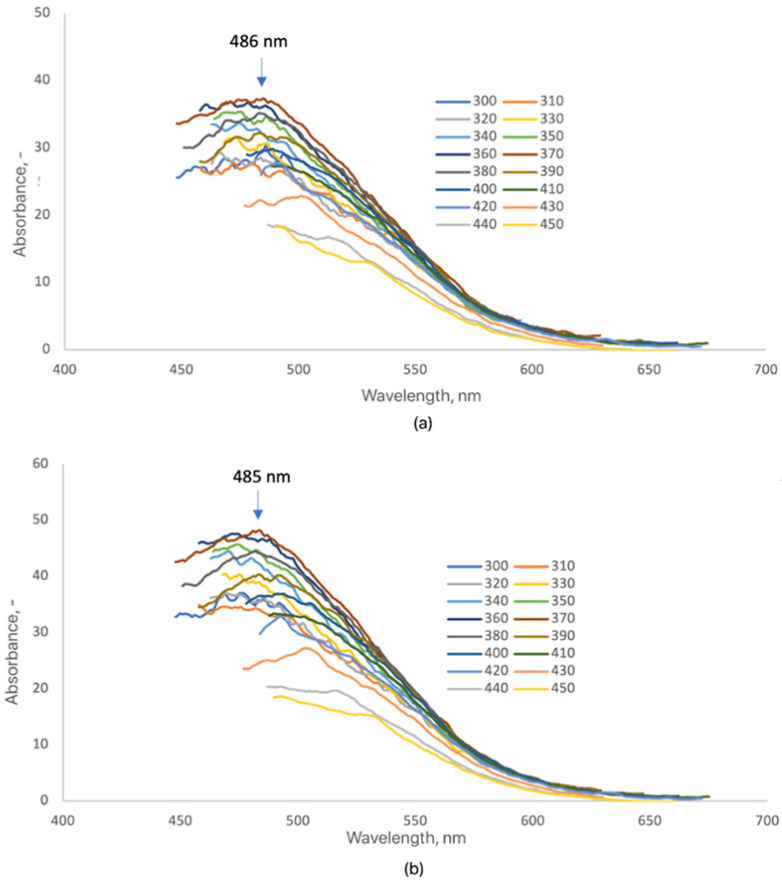
Fluorescence emission spectra of the samples (3 mg/mL): (**a**) CQDs-5-Cum; (**b**) CQDs-6-Cum (each color represents a different excitation wavelength).

The surface modification of CQDs-5 and CQDs-6 with 7-amino-4-(trifluoromethyl)coumarin caused a distinct alteration in their fluorescence behavior (Figure 9). Unlike the previously analyzed samples (CQDs-2 and CQDs-3), both CQDs-5-Cum and CQDs-6-Cum displayed a single, well-defined emission maximum at 486 nm and 485 nm, respectively. The appearance of a single dominant band suggests that the coumarin chromophore dictates the emissive behavior through strong π–π interactions and efficient energy transfer between the coumarin ring and the CQD core [16,20,25,50]. A marked enhancement in fluorescence intensity compared with unmodified CQDs confirms successful surface functionalization and improved radiative recombination efficiency [47,48]. The observed redshift in emission relative to pristine CQDs indicates extended conjugation and new emissive states introduced by coumarin coupling, consistent with previous reports on dye-sensitized or heteroatom-modified CQDs [25,48,50,52]. Despite the spectral shift, the excitation-dependent emission typical for carbon quantum dots was retained, demonstrating that the intrinsic electronic structure and quantum confinement of the CQD core remained intact. These results confirm that coumarin doping effectively enhances the optical performance of CQDs while preserving their fundamental photophysical properties, supporting their potential use in bioimaging and optoelectronic applications [6,16,20,25,47].

**Figure 10:**
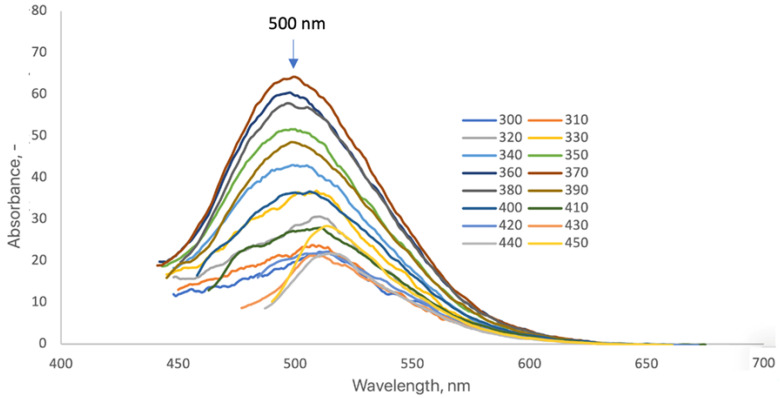
Fluorescence emission spectra of the CQDs-8-Cum sample (3 mg/mL) (each color represents a different excitation wavelength).

The modification of CQDs-8 with 7-amino-4-(trifluoromethyl)coumarin produced a strong enhancement and a distinct redshift of the fluorescence emission, with a single, well-defined maximum centered at 500 nm (Figure 10). The broad emission band, spanning approximately 460–550 nm, intensified with increasing excitation wavelengths (300–410 nm), confirming the excitation-dependent behavior typical of carbon quantum dots [47,48]. The bathochromic shift relative to unmodified CQDs indicates strong π–π and electronic coupling between the coumarin chromophore and the conjugated domains of the CQD surface [16,20,25,50]. The marked increase in emission intensity reflects efficient energy transfer from the excited coumarin states to the CQD core, consistent with the dye’s role as a light-harvesting antenna that enhances radiative recombination efficiency [47,48,52]. Overall, the results confirm that coumarin functionalization effectively shifts CQD emission toward the green spectral region while maintaining its intrinsic excitation-dependent photoluminescence and quantum-confined structure. Such spectral tuning demonstrates the strong potential of coumarin-modified CQDs for bioimaging and optoelectronic applications [6,16,20,25,50].

### 2.5. TEM Imaging

**Figure 11:**
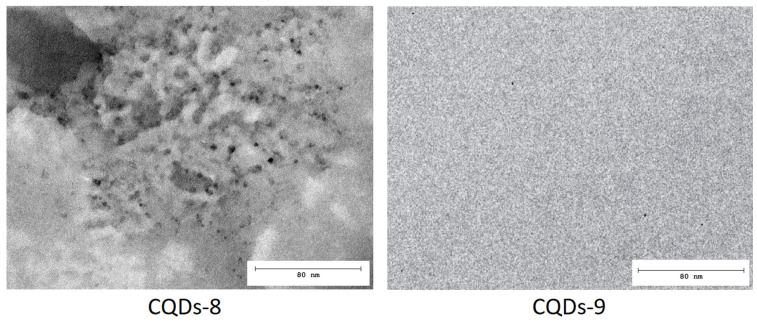
TEM microphotograph of the CQDs-8 and CQDs-9 sample.

**Figure 12:**
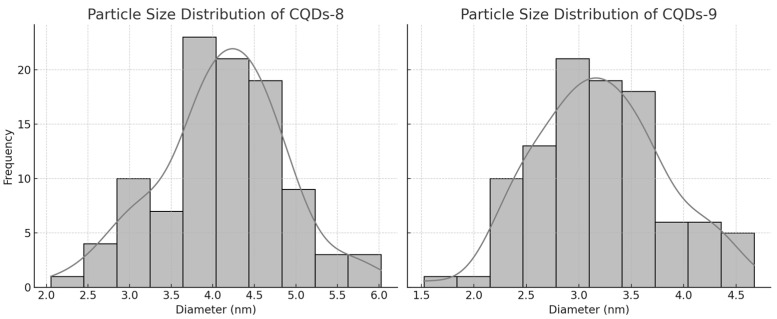
Histograms of the CQDs-8 and CQDs-9 size distribution.

Transmission electron microscopy (TEM) images of dopamine-modified CQDs-8 and CQDs-9 revealed uniformly dispersed, quasi-spherical nanoparticles with well-defined contrast typical of carbonaceous nanostructures (Figure 11). The CQDs-8 micrograph showed moderately dispersed nanodots with minor aggregation, likely resulting from incomplete nucleation at shorter synthesis duration. In contrast, CQDs-9 exhibited a more homogeneous morphology composed of uniformly distributed spherical particles with smaller average diameters, indicating that prolonged microwave irradiation (5 min) promoted more complete carbonization and improved size uniformity (Figure 12). [16,20,47].

The estimated particle diameters ranged between 2 and 6 nm, consistent with previously reported values for microwave-synthesized CQDs [6,47,48]. High-contrast regions within the particles suggest the presence of partially graphitized domains embedded in an amorphous carbon matrix, typical for CQDs derived from organic precursors [25,50]. The absence of significant aggregation confirms good colloidal stability conferred by dopamine surface modification, which likely enhances dispersion and fluorescence performance [20,25].

Overall, the TEM analysis confirmed the formation of uniform, nanometer-scale CQDs with partially crystalline order and strong structural integrity, suitable for bioimaging, biosensing, and optoelectronic applications, where high dispersibility and stable emission are essential [6,16,20,25,47].

### 2.6. Cytotoxicity Assessment

**Figure 13:**
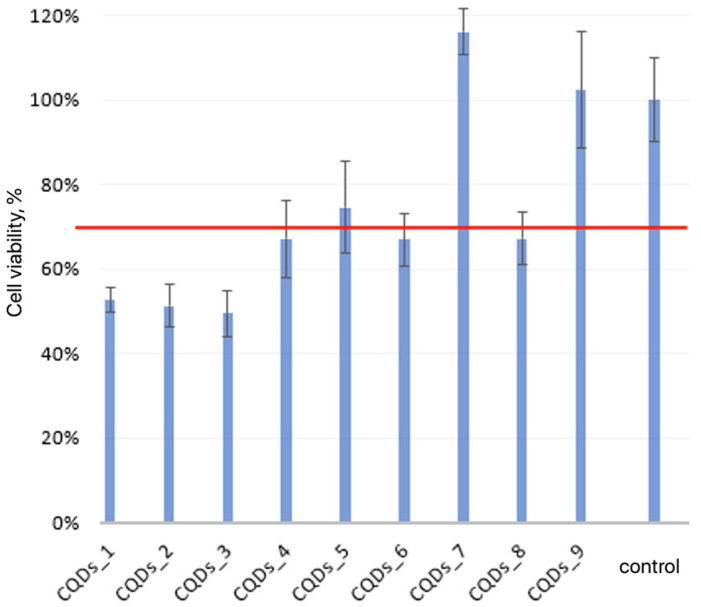
Quantitative viability assessment on MG-63 osteosarcoma cell line (red line represents the cytotoxicity limit; error bars indicate standard deviation (n = 5)).

**Figure 14:**
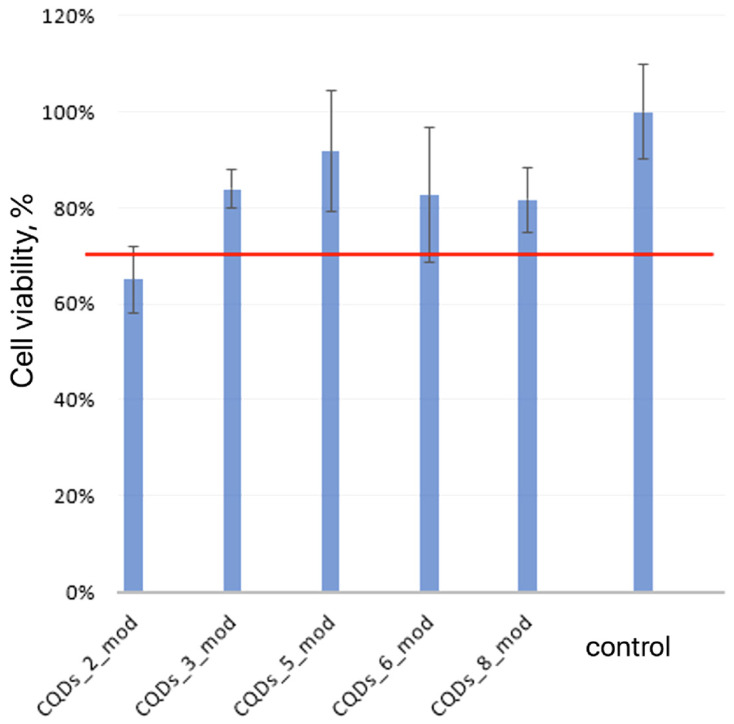
Quantitative viability assessment on MG-63 osteosarcoma cell line for modified samples (red line represents the cytotoxicity limit; error bars indicate standard deviation (n = 5)).

The cytotoxicity of the synthesized CQDs was evaluated using the XTT assay on osteosarcoma MG-63 cells, following the ISO 10993-5:2009 standard Biological evaluation of medical devices Part 5: Tests for in vitro cytotoxicity, 3^rd^ edition, Geneve, Switzerland, 2009. which defines cell viability ≥70% as the threshold for non-cytotoxicity. The assay was performed by adding 0.05 cm^3^ of CQD suspension to 0.25 cm^3^ of cell culture medium, and the results are presented in Figure 13 and Figure 14. Among the tested samples, CQDs-5, CQDs-7, and CQDs-9 (before modification) and CQDs-3, CQDs-5, CQDs-6, and CQDs-8 (after modification) demonstrated cell viabilities above 70%, confirming non-cytotoxic behavior in accordance with ISO 10993-5. The remaining samples showed reduced viability, indicating potential cytotoxic effects at the tested concentration. The variation in cytotoxicity is attributed to differences in surface chemistry and functionalization. Surface passivation with biomolecules such as collagen or dopamine improves biocompatibility by reducing reactive oxygen species and limiting oxidative stress, consistent with earlier studies on biogenic and surface-modified CQDs used for cellular imaging and biosensing [16,20,22,47,50].

Overall, these findings demonstrate that the biological response of MG-63 cells is strongly influenced by synthesis parameters and surface modification. The non-toxic, functionalized CQDs exhibit excellent potential for bioimaging, biosensing, and circulating tumor cell (CTC) detection, where low cytotoxicity and high photostability are crucial [31,35,47,50,60].

**Figure 15:**
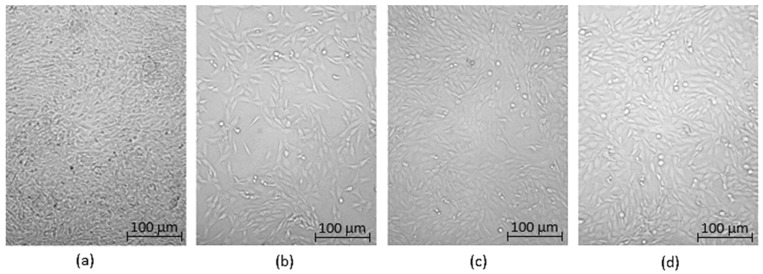
Qualitative viability assessment on MG-63 osteosarcoma cell line at different concentrations: (**a**) 0.05 cm^3^; (**b**) 0.1 cm^3^; (**c**) 0.15 cm^3^; (**d**) 0.2 cm^3^ CQDs-1 sample.

**Figure 16:**
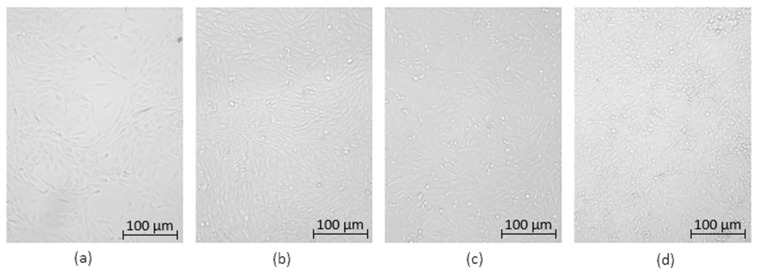
Qualitative viability assessment on MG-63 osteosarcoma cell line at different concentrations: (**a**) 0.05 cm^3^; (**b**) 0.1 cm^3^; (**c**) 0.15 cm^3^; (**d**) 0.2 cm^3^ CQDs-2 sample.

**Figure 17:**
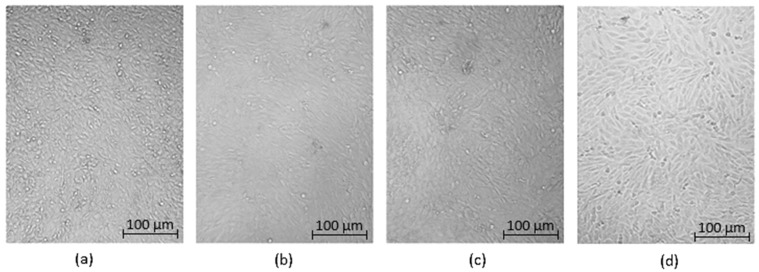
Qualitative viability assessment on MG-63 osteosarcoma cell line at different concentrations: (**a**) 0.05 cm^3^; (**b**) 0.1 cm^3^; (**c**) 0.15 cm^3^; (**d**) 0.2 cm^3^ CQDs-3 sample.

**Figure 18:**
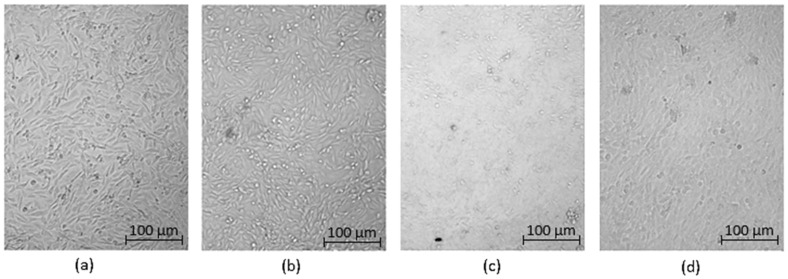
Qualitative viability assessment on MG-63 osteosarcoma cell line at different concentrations: (**a**) 0.05 cm^3^; (**b**) 0.1 cm^3^; (**c**) 0.15 cm^3^; (**d**) 0.2 cm^3^ CQDs-4 sample.

**Figure 19:**
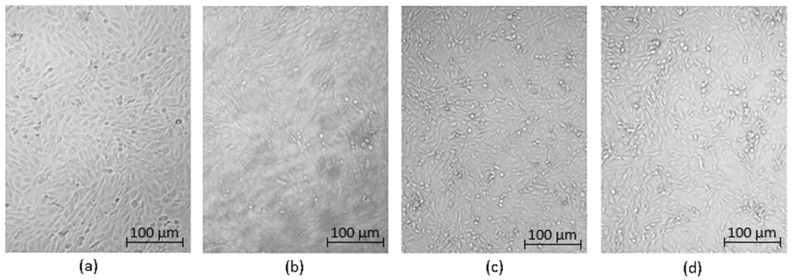
Qualitative viability assessment on MG-63 osteosarcoma cell line at different concentrations: (**a**) 0.05 cm^3^; (**b**) 0.1 cm^3^; (**c**) 0.15 cm^3^; (**d**) 0.2 cm^3^ CQDs-5 sample.

**Figure 20:**
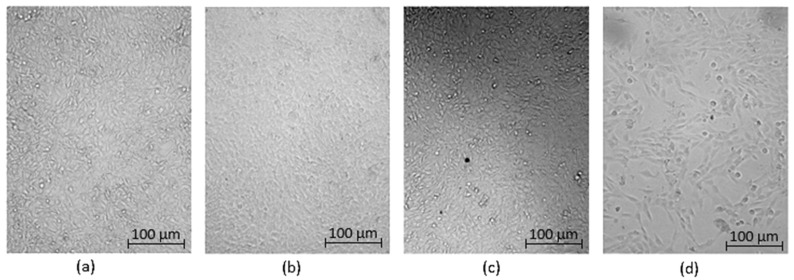
Qualitative viability assessment on MG-63 osteosarcoma cell line at different concentrations: (**a**) 0.05 cm^3^; (**b**) 0.1 cm^3^; (**c**) 0.15 cm^3^; (**d**) 0.2 cm^3^ CQDs-6 sample.

**Figure 21:**
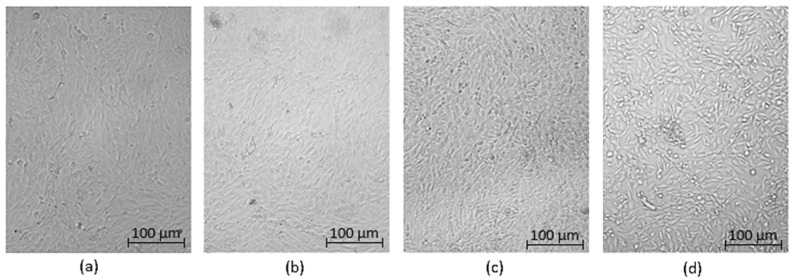
Qualitative viability assessment on MG-63 osteosarcoma cell line at different concentrations: (**a**) 0.05 cm^3^; (**b**) 0.1 cm^3^; (**c**) 0.15 cm^3^; (**d**) 0.2 cm^3^ CQDs-7 sample.

**Figure 22:**
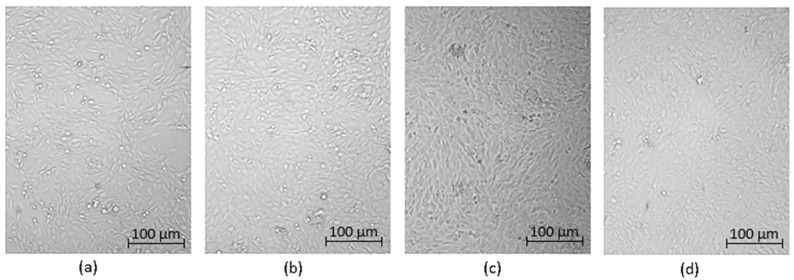
Qualitative viability assessment on MG-63 osteosarcoma cell line at different concentrations: (**a**) 0.05 cm^3^; (**b**) 0.1 cm^3^; (**c**) 0.15 cm^3^; (**d**) 0.2 cm^3^ CQDs-8 sample.

**Figure 23:**
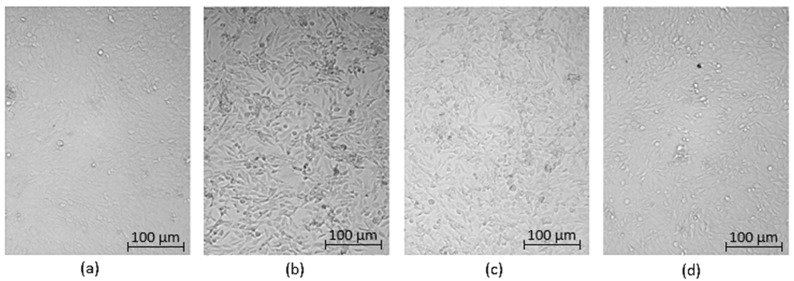
Qualitative viability assessment on MG-63 osteosarcoma cell line at different concentrations: (**a**) 0.05 cm^3^; (**b**) 0.1 cm^3^; (**c**) 0.15 cm^3^; (**d**) 0.2 cm^3^ CQDs-9 sample.

Optical microscopy was used to qualitatively assess the cytotoxicity of CQDs toward osteosarcoma MG-63 cells at different concentrations (0.05–0.2 cm^3^ per 0.25 cm^3^ of cell culture medium), as shown in Figure 15, Figure 16, Figure 17, Figure 18, Figure 19, Figure 20, Figure 21, Figure 22 and Figure 23. The analysis revealed that, for most samples, the cells maintained normal spindle-shaped morphology, high confluence, and clear cytoplasmic boundaries, indicating good biocompatibility and the absence of major cytotoxic effects. For CQDs-1–5, no morphological abnormalities were observed across all tested concentrations, with only a few isolated dead cells detected at the highest doses. Similarly, CQDs-6–9 preserved typical morphology and high confluence, confirming that surface modification, particularly with collagen and dopamine, did not induce visible cellular damage. Minor decreases in cell density observed at higher concentrations of CQDs-6 suggest a concentration-dependent response, yet still within the non-cytotoxic threshold defined by ISO 10993-5:2009 [16,20,22].

The consistent spindle-like morphology and absence of membrane disruption or rounding indicate that the CQDs did not interfere with cell adhesion or proliferation, supporting earlier findings on the biocompatibility of nitrogen- and biomolecule-doped carbon dots [47,48,50]. These results align with the XTT assay data, confirming that the tested CQDs exhibit low cytotoxicity and are suitable for biological applications.

**Figure 24:**
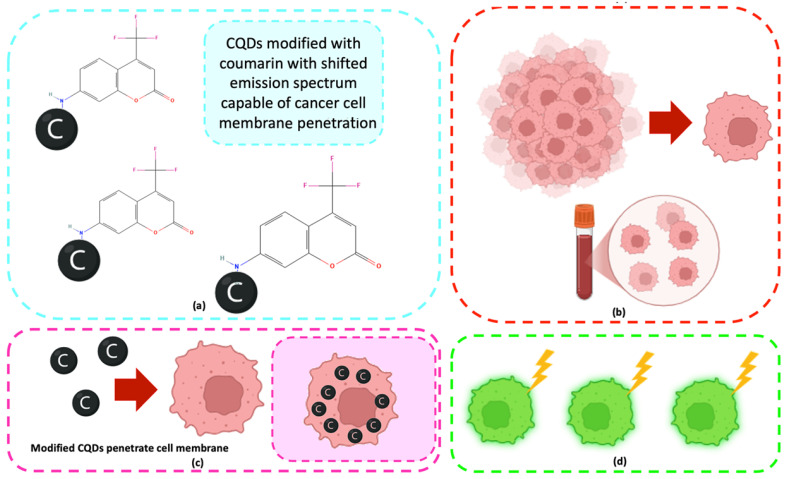
(**a**) Obtainment of surface-modified CQDs; (**b**) collection of blood sample containing circulating tumor cells; (**c**) incubation; (**d**) fluorescence emission of tumor cells.

Overall, the microscopy analysis supports the potential use of these CQDs in bioimaging and circulating tumor cell (CTC) detection, where minimal cytotoxicity and stable interaction with living cells are essential (Figure 24) [31,35,50,60].

### 2.7. In Vitro Bioimaging

**Figure 25:**
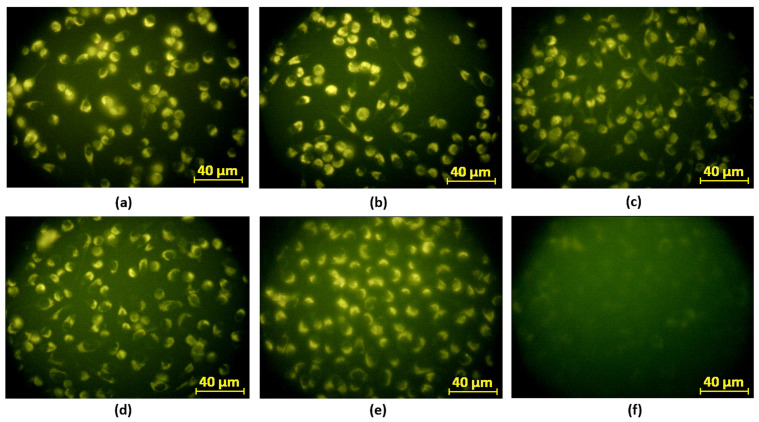
Fluorescence images of astrocytoma 1321N1 cells treated with different CQDs (excitation: 360 nm). Fluorescent emission is visible in cells incubated with (**a**) CQDs-2, (**b**) CQDs-3, (**c**) CQDs-5, (**d**) CQDs-6, (**e**) CQDs-8, and (**f**) CQDs-9. Strong intracellular staining is observed in (**a**,**b**,**e**), indicating efficient uptake and preserved luminescent properties (0.2 cm^3^).

The in vitro fluorescence imaging of astrocytoma 1321N1 cells was conducted using an inverted epifluorescence microscope to evaluate the potential of various CQD samples for bioimaging applications (Figure 25). The tested samples included CQDs-2, CQDs-3, CQDs-5, CQDs-6, and CQDs-8. The fluorescence intensity and image quality were assessed for each (Figure 24). Among these, CQDs-2, CQDs-3, and CQDs-8 demonstrated the most effective fluorescence labeling of cells, exhibiting bright, uniform emission and high image contrast. The cells treated with these CQDs showed clearly defined morphologies and consistent fluorescence intensity, suggesting efficient cellular uptake and minimal aggregation. Such properties are crucial for reliable bioimaging and have been similarly observed in nitrogen- and dopamine-modified CQDs with enhanced photoluminescence [48,50,51,60]. In contrast, CQDs-5 and CQDs-6 produced weaker fluorescence and lower contrast, likely due to less effective surface passivation or reduced quantum yield, consistent with previous findings linking CQD surface chemistry to emission efficiency [49,52]. The strong, stable fluorescence of CQDs-2, CQDs-3, and CQDs-8 highlights their potential as fluorescent probes for cancer diagnostics, particularly for detecting and monitoring astrocytoma cells. The emission stability and high photoluminescence intensity of these CQDs support their suitability for real-time cellular imaging and tracking applications [50,51,55,60].

In conclusion, CQDs-2, CQDs-3, and CQDs-8 displayed the best fluorescence performance and imaging contrast, making them promising candidates for future research in glioma imaging and diagnostics. Further studies should aim to optimize surface functionalization to improve tumor-specific targeting and enhance imaging selectivity [48,50,56].

## 3. Discussion

When carbon quantum dots (CQDs) enter biological environments, they rapidly interact with biomolecules such as proteins, lipids, and nucleic acids, forming a dynamic and complex layer known as the biomolecular corona. This corona effectively redefines the nanoparticle’s biological identity, influencing how it is recognized and processed by cells, and can modulate colloidal stability, biodistribution, and fluorescence behavior [11,13,20,22].

In the context of our study, although we did not directly investigate corona formation, the functionalization of CQDs with biomolecules such as betaine, marine collagen, dopamine, and 7-amino-4-(trifluoromethyl)coumarin introduced various surface moieties—including amine, hydroxyl, carboxyl, and aromatic groups—that are known to favor non-covalent interactions with serum proteins. This strongly suggests that corona formation likely occurred during in vitro experiments, particularly during incubation with culture medium containing fetal bovine serum (FBS), as supported by the literature on similar nanostructures [15,20,22,29].

The impact of this biomolecular corona may explain some of the differences in fluorescence intensity and cellular uptake efficiency observed among CQD variants. For example, samples such as CQDs-2, CQDs-3, and CQDs-8 exhibited enhanced intracellular fluorescence and better cell imaging contrast, which could be attributed not only to their intrinsic surface chemistry but also to the nature and composition of the protein corona formed around them. In contrast, CQDs-5 and CQDs-6, which showed weaker fluorescence responses, may have formed coronas that hindered effective interaction with the cellular membrane or interfered with fluorescence emission. Moreover, dopamine- and collagen-functionalized CQDs demonstrated superior biocompatibility in XTT assays, suggesting that their coronas may have shielded reactive surface sites or facilitated better integration with cell membranes, reducing cytotoxicity.

Understanding corona-mediated modulation of CQD behavior is particularly important for potential applications in circulating tumor cell (CTC) detection. In such applications, the selectivity and targeting efficiency of nanoprobes can be compromised or enhanced depending on the biomolecular corona’s composition. For instance, opsonins in the corona may lead to nonspecific uptake by immune cells, whereas the presence of certain adhesion or receptor-binding proteins may enhance interaction with tumor-derived cells [13,22,31,35].

To better characterize these effects, future studies should include proteomic profiling (e.g., LC–MS/MS, Western blotting, or label-free biosensor analyses) of the protein corona formed on differently modified CQDs. Such data would allow correlation between corona composition and observed bioimaging or cytotoxicity behavior, enabling more predictive and rational design of surface-functionalized CQDs tailored to specific diagnostic purposes [11,22,60].

In summary, while the current study did not directly characterize corona formation, the observed differences in fluorescence performance and cell viability among CQD variants suggest that surface interactions with biomolecules play a critical role in determining their biological behavior. A deeper understanding of how specific surface modifications influence corona composition will be essential for optimizing CQDs for future biomedical applications, including cancer cell imaging and circulating tumor cell detection.

## 4. Materials and Methods

### 4.1. Materials

The following materials were used in this study: glucosamine, glucose, coconut betaine, collagen, dopamine, 7-amino-4-trifluoromethylcoumarin (97%), and osteosarcoma MG-63 cells, as well as astrocytoma 1321N1 cells (European Collection of Authenticated Cell Cultures (ECACC) Porton Down, Salisbury, Great Britain), all purchased from Sigma-Aldrich (St. Louis, MO, USA). Hydrochloric acid (37%) and sodium hydroxide (10%) were obtained from Chempur (Piekary Śląskie, Poland). Fetal bovine serum (FBS) and DMEM medium (high glucose, with L-glutamine) were also sourced from Sigma-Aldrich (St. Louis, MO, USA). Penicillin–streptomycin solution (100×) was purchased from Sigma-Aldrich (St. Louis, MO, USA). XTT tetrazolium salt was obtained from Roche (Basel, Switzerland).

### 4.2. Methods

#### 4.2.1. CQD Synthesis and Modification

The raw materials used as a carbon-rich precursor for the synthesis of carbon quantum dots included glucosamine. The additives used to modify the properties of CQDs were coconut betaine, collagen, and dopamine. Table 2 provides a detailed list of the raw materials used in the CQD synthesis process and the reaction times.

Nine vessels were weighed on an analytical balance with 1 g of glucosamine each. To three samples of glucosamine, 0.1 g of betaine was added, to another three samples, 0.1 g of collagen, and to the remaining samples, 0.05 g of dopamine. The following steps for each sample were the same: the raw material, along with the additives, was placed in a crucible, and 3 cm^3^ of distilled water and 1 cm^3^ of hydrochloric acid (serving as a catalyst) were added. The crucible was then exposed to a microwave radiation field with a power of 800 W. Three samples with each additive were prepared to carry out the process at different time intervals of 3, 4, and 5 min.

The next step involved transferring the charred mixture containing CQDs into a beaker and adding 15–25 cm^3^ of water. Each sample was placed in an ultrasonic bath for 5 min and then neutralized with a 10% NaOH solution to pH = 7, preventing degradation of the dialysis membrane. The solutions were filtered through syringe membrane filters to separate the carbon microparticles from the CQDs. The resulting filtrates were transferred into dialysis tubes made of regenerated cellulose (MWCO = 1000 Da). The dialysis lasted 3 days; the water was changed every 24 h. The entire setup was placed in beakers filled with 500 cm^3^ of distilled water to separate the CQDs from low-molecular-weight byproducts, such as levulinic acid, furfural, dyes, and unreacted components from the reaction mixture. The selected samples were then surface-modified with a solution of 7-amino-4-trifluoromethylcoumarin in the presence of EDC (1-ethyl-3-[3-dimethylaminopropyl]carbodiimide) as a coupling agent, facilitating a reaction between the amine group of the coumarin ring and the carboxyl groups on the surface of the CQDs. Their absorbance and fluorescence were then re-measured.

#### 4.2.2. UV–Vis Spectroscopy of CQDs and Modified CQDs

The UV–Vis absorption spectra of both carbon quantum dots (CQDs) and their modified forms were recorded using an Agilent 8453 spectrophotometer (Agilent Technologies, Santa Clara, CA, USA) equipped with a diode array. The measurements were performed in a quartz cuvette with a 1 cm optical path length, covering a wavelength range from 200 to 800 nm. The CQDs and their modified versions were dissolved in water prior to measurement. Spectra were obtained in triplicate to ensure consistency and reproducibility. Shifts and variations in the absorption peaks were analyzed to evaluate the impact of modification on the optical properties of the CQDs. For the measurements, aqueous solutions of 3 mg/mL concentrations of different amounts were used (0.05 cm^3^; 0.1 cm^3^; 0.15 cm^3^, and 0.2 cm^3^).

#### 4.2.3. Fluorescence Measurement of Carbon Quantum Dot (CQD) Solutions

Fluorescence measurements of the carbon quantum dot (CQD) solutions were performed using a JASCO FP-750 spectrofluorometer (JASCO, Tokyo, Japan). Optical slits of 5 nm were used for both the excitation and emission wavelengths, as well as for the radiation reaching the detector. The scanning speed was set to 200 nm/min to ensure precise spectral data. Fluorescence spectra were recorded for a CQD solution with a concentration corresponding to an absorbance of A = 0.100 at an excitation wavelength of 365 nm. The solutions were prepared in water and analyzed in a suitable cuvette. Spectra were recorded in triplicate to ensure reproducibility and reliability of the results. For the measurements, aqueous solutions of 3 mg/mL concentrations were used.

#### 4.2.4. FT-IR Analysis

FT-IR analysis was performed using a Nicolet Nexus 470 spectrometer (Thermo Scientific, Waltham, MA, USA) equipped with a diamond ATR attachment. The spectral resolution was set to 4 cm^−1^, and 32 scans were conducted for each sample. To prepare the samples for analysis, CQD solutions were repeatedly dropped onto polystyrene plates to form a thin layer suitable for measurement. Each time, drops of aqueous solutions of 3 mg/mL concentrations were instilled and left to dry.

#### 4.2.5. TEM Imaging of CQD Samples

Transmission electron microscopy (TEM) images of the carbon quantum dots (CQDs) were acquired using a JEOL JEM 2100 transmission electron microscope (JEOL Ltd., Tokyo, Japan) with a magnification of 30,000×. For imaging, a 5 µL drop of CQD solution was placed on a carbon-coated copper grid. The solvent was allowed to evaporate, leaving the CQDs on the grid surface using aqueous solution of 3 mg/mL concentration. After drying, the grid was placed in the TEM chamber, and high-resolution images were recorded to visualize the morphology and size of the CQDs.

#### 4.2.6. Bright-Field and Fluorescence Microscopic Cell Observations

Cell observations were performed using a Delta Optical IB-100 LED inverted microscope (Delta Optical, Mińsk Mazowiecki, Poland). The microscope is equipped with a built-in fluorescence attachment, allowing for both bright-field and fluorescence imaging without the need for additional accessories. The excitation wavelength of 470 nm was used for fluorescence imaging. The cells were incubated under appropriate conditions and then exposed to the specific excitation wavelength of 470 nm. Images were captured using the integrated camera, enabling high-quality images of the cells. Bright-field images were also recorded for comparative analysis. The imaging was conducted at magnifications of 200× and 400×.

#### 4.2.7. Qualitative and Quantitative Assessment on Osteosarcoma MG-63 Cell Line

The human osteosarcoma cell line MG-63 (Sigma-Aldrich, St. Louis, MO, USA). The cells were cultured in Dulbecco’s Modified Eagle Medium (DMEM, Gibco, Thermo Fisher Scientific, Waltham, MA, USA) supplemented with 10% fetal bovine serum (FBS, Sigma-Aldrich, St. Louis, MO, USA) and 1% penicillin–streptomycin (Gibco, Thermo Fisher Scientific). The cells were maintained at 37 °C in a humidified atmosphere containing 5% CO_2_ and passaged using 0.25% trypsin–EDTA (Gibco, Thermo Fisher Scientific) when they reached 80–90% confluence. In order to assess the qualitative cytotoxicity, CQD nanomaterials were sterilized using syringe filters. All samples were placed in 24-well plates. Then, 1 cm^3^ of osteosarcoma MG-63 cell solution was added to each well. The prepared plates were incubated for 48 h at 37 °C in a CO_2_ incubator (S-Bt, Smart Biotherm, SIA Biosan, Riga, Latvia). After 48 h, cell evaluation was performed using an inverted microscope.

The quantitative cytotoxicity of the tested samples was evaluated using the XTT assay. For this purpose, cells were seeded in a 96-well plate at a density of 1 × 10^4^ cells per well and allowed to attach overnight. The next day, the culture medium was replaced with fresh medium containing different concentrations of the test samples, while untreated cells served as a negative control. After 48 h of incubation, the XTT reagent was prepared according to the manufacturer’s instructions and added to each well, followed by further incubation for 4 h in the dark. The absorbance of the formazan product was measured at 450 nm with a reference wavelength of 630 nm using a microplate reader. Cell viability was calculated as a percentage relative to the untreated control group. All measurements were performed with 5 repetitions.

After 48 h, the cells were examined under an inverted phase-contrast microscope to qualitatively evaluate morphological changes indicative of cytotoxic effects. The cytotoxic response was assessed based on characteristic alterations such as cell rounding or shrinkage, detachment from the substrate, cytoplasmic granulation, nuclear condensation, and loss of confluence. The degree of cytotoxicity was classified semi-quantitatively according to the percentage of affected cells in comparison with negative (untreated cells) and positive (e.g., phenol-treated) controls, following the ISO 10993-5 qualitative grading scale. According to this scale, Grade 0 corresponds to no cytotoxicity (no morphological changes), Grade 1 to slight cytotoxicity (<20% affected cells), Grade 2 to mild cytotoxicity (20–50% affected cells), Grade 3 to moderate cytotoxicity (50–70% affected cells), and Grade 4 to severe cytotoxicity (>70% affected cells).

#### 4.2.8. *In Vitro* Bioimaging on 1321N1 Cell Line

In vitro bioimaging of astrocytoma 1321N1 cells was performed using an inverted microscope equipped with an epifluorescence adapter. The cells were seeded at a density of 1 × 10^4^ cells per well in a 24-well plate and allowed to adhere overnight in DMEM supplemented with 10% fetal bovine serum (FBS) and 1% penicillin–streptomycin. After 24 h, the medium was replaced with fresh medium containing sterile solutions of carbon quantum dots (CQDs) at varying concentrations. The cells were incubated with CQDs for 2 h at 37 °C in a humidified incubator with 5% CO_2_. After incubation, the cells were washed with phosphate-buffered saline (PBS) to remove any unbound CQDs and then, the cells were washed again with PBS and then visualized using the inverted microscope with epifluorescence. Fluorescence images were captured under appropriate excitation and emission wavelengths specific to the CQDs’ fluorescence characteristics. Cell morphology and fluorescence intensity were assessed to determine the cellular uptake and distribution of CQDs.

## 5. Conclusions

In this study, carbon quantum dots (CQDs) were successfully synthesized from natural precursors using a microwave-assisted approach, with glucosamine as a carbon source and betaine, marine collagen, and dopamine as surface modifiers. Subsequent functionalization with 7-amino-4-(trifluoromethyl)coumarin significantly enhanced the fluorescence properties and altered the surface chemistry of the obtained nanomaterials.

Spectroscopic analyses confirmed the presence of characteristic surface groups and well-developed π-conjugated domains, while fluorescence studies revealed strong, excitation-dependent emission in the blue-to-green region. The modified CQDs exhibited excellent aqueous stability and high photoluminescence efficiency, indicating their suitability as fluorescent nanoprobes.

Cytotoxicity assays on MG-63 osteosarcoma cells demonstrated that several CQD variants were non-toxic and biocompatible, particularly those modified with collagen and dopamine. Moreover, fluorescence microscopy confirmed that selected CQDs (CQDs-2, CQDs-3, and CQDs-8) provided clear visualization of astrocytoma 1321N1 cells, confirming their effective cellular uptake and strong emission properties.

The synthesized CQDs demonstrated favorable fluorescence and biocompatibility profiles, supporting their potential for future development in biomedical imaging and diagnostic applications, including the detection of circulating tumor cells. However, further investigation is required to assess their specificity and performance in more complex biological systems. Future work should focus on optimizing surface functionalization and targeting mechanisms to enhance the selectivity and clinical applicability of CQDs in oncology.

## Figures and Tables

**Table 1:** Summary of the state of the art.

Study (Year)	CQD Source/Modification	Application Target	Emission/(QY)	Cells/CTC Context	Key Contribution
Liu et al., ACS Angew. Chem. Int. Ed (2007) [46]	Candle soot–derived CQDs (nitric acid–oxidized)	Fluorescent labeling and biosensing.	Multicolor emission (415–615 nm)	-	Simple synthesis of multicolor fluorescent CQDs from candle soot using a low-cost carbon source.
Pal et al., ACS Omega (2018) [47]	Curcumin-derived, PEI-passivated CQDs.	Multicolor bioimaging and biolabeling	Multicolor (blue–green–red)	Tested in cancer (A549, HCT-15) and normal (NIH 3T3) cells;	Green synthesis of PEI-passivated, multicolor CQDs from curcumin with in vitro and in vivo bioimaging capability.
Liu et al., Sci. Rep. (2018) [48]	Folic acid–derived, nitrogen-doped CQDs synthesized by one-step hydrothermal treatment.	Targeted fluorescence imaging of folate receptor–positive cancer cells.	Blue emission (~400 nm)	Tested on folate receptor–positive cancer cells (HeLa, SKOV-3) and FR-negative A549 cells;	One-step synthesis of ultrabright, folate-targeting CQDs with record-high QY enabling selective imaging of FR-positive cancer cells.
Al-Hetty et al., Inorg. Chem. Commun (2022) [28]	CQDs from diverse chemical and biomass precursors, engineered via heteroatom doping and surface functionalization (review).	Cancer bioimaging (in vitro and in vivo), including targeted tumor imaging and imaging-guided therapy.	Tunable emission from blue to NIR	In vitro and in vivo cancer cell and tumor models.	omprehensive review of CQD/GQD engineering strategies enabling targeted cancer bioimaging and imaging-guided therapy.
Anpalagan et al., Nanomaterials (2023) [50]	Bread-derived CQDs (green synthesis)	Bioimaging of colon cancer cells (CT-26 and HT-29)	Green fluorescence (~540 nm)	Tested on mouse (CT-26) and human (HT-29) colon cancer cells	Demonstration of a rapid, chemical-free, low-energy synthesis of biocompatible CQDs from bread for cancer cell bioimaging.
Latha et al., Biomed. Eng. Adv (2023) (review) [51]	Various biomass- and chemically derived CQDs with surface/dopant modifications.	Tumor diagnosis	Tunable visible–NIR fluorescence with reported QY values up to ~94%	Cancer cells (in vitro)	Systematic review of CQDs for cancer imaging and diagnosis.
Bhattacharya et al., Pharmaceutics (2023) [52]	Diverse CQDs from chemical precursors and biowaste with surface functionalization/doping	Targeted imaging and therapy of breast, cervical, lung, liver, and brain cancers.	Tunable blue–NIR emission with reported QY values ranging from a few % up to >90%	In vitro and in vivo studies on multiple cancer cell lines and tumor models;	Comprehensive review of CQDs as multifunctional nanotheranostic agents for cancer imaging and therapy.
Murugan B. et al., J. Pharm. Biomed. Anal. Open (2025) [53]	CQDs synthesized from de-oiled copra cake biowaste via one-pot hydrothermal carbonization	Biocompatible fluorescence bioimaging in biological systems.	Blue fluorescence with excitation-independent emission;	General biological cell and organism imaging models	Demonstration of a green, biowaste-derived CQD platform for biocompatible fluorescence bioimaging.
Cui et al., Talanta (2019) [54]	QD-labeled magnetic platform	CTC detection in clinical samples	Bright, narrowband QD emission for detection	CTCs (model/clinical)	Clinical validation of an opto-magnetic approach to CTC detection.
Wang. H. et al. Bioact. Mater,. (2024) [55]	Carbon dots, graphene quantum dots, and polymer dots derived from chemical or biomass precursors with surface functionalization and heteroatom doping (review).	Tumor diagnosis via fluorescence imaging and detection of cancer hallmarks (tumor tissues and biomarkers).	Tunable fluorescence from UV–visible to NIR	In vitro and in vivo tumor cell models; includes CTC-related biomarker detection (reviewed studies).	Framework linking CQD structure–function design to cancer hallmarks for improved tumor diagnosis.
Kim et al., J. Hematol. Oncol. (2024) (review) [56]	Carbon dots from chemical or biomass precursors with surface functionalization/doping.	Optical detection of liquid biopsy biomarkers (CTCs, ctDNA, proteins, exosomes)	Broad tunable fluorescence (UV–Vis–NIR)	CTC detection and analysis in liquid biopsy	Systematic comparison of optical nanomaterial strategies (including carbon dots) for ultrasensitive detection of CTCs, ctDNA, proteins, and exosomes in liquid biopsy.
Chai, L et al., ACS Appl. Mater. Interfaces 2015) (review) [57]	Activated carbon–derived CQDs chemically oxidized and covalently functionalized with dopamine (Dopa-CQDs)	Tyrosinase activity monitoring, inhibitor screening, and intracellular imaging (melanoma cells).	Blue emission (~425 nm)	Tested in melanoma (B16) and HeLa cells	Development of dopamine-functionalized CQDs enabling real-time intracellular tyrosinase sensing and inhibitor screening via PET-based fluorescence quenching.
Unnikrishnan et al., ACS Omega (2020) [58]	Various CDs; organelle-/target-directed functionalization	Selective subcellular labeling	Visible emission enhanced by coumarin	L929 fibroblasts (live imaging)	Mechanistic basis: how functional groups/bioligands enable specificity supporting surface modification selection

**Table 2:** Sample description.

Sample	Glucosamine, g	Betaine, g	Marine Collagen, g	Dopamine Hydrochloride, g	Reaction Time, min
CQDs-1	1.00	0.10	0.0	0.0	3
CQDs-2					4
CQDs-3					5
CQDs-4		0.0	0.1		3
CQDs-5					4
CQDs-6					5
CQDs-7			0.0	0.05	3
CQDs-8					4
CQDs-9					5

## Data Availability

Data available on request.
